# Role of Inflammatory Cytokines in COVID-19 Patients: A Review on Molecular Mechanisms, Immune Functions, Immunopathology and Immunomodulatory Drugs to Counter Cytokine Storm

**DOI:** 10.3390/vaccines9050436

**Published:** 2021-04-29

**Authors:** Ali A. Rabaan, Shamsah H. Al-Ahmed, Javed Muhammad, Amjad Khan, Anupam A Sule, Raghavendra Tirupathi, Abbas Al Mutair, Saad Alhumaid, Awad Al-Omari, Manish Dhawan, Ruchi Tiwari, Khan Sharun, Ranjan K. Mohapatra, Saikat Mitra, Muhammad Bilal, Salem A. Alyami, Talha Bin Emran, Mohammad Ali Moni, Kuldeep Dhama

**Affiliations:** 1 Molecular Diagnostic Laboratory, Johns Hopkins Aramco Healthcare, Dhahran 31311, Saudi Arabia; ali.rabaan@jhah.com; 2 Specialty Paediatric Medicine, Qatif Central Hospital, Qatif 32654, Saudi Arabia; shalahmed@moh.gov.sa; 3 Department of Microbiology, The University of Haripur, Khyber Pakhtunkhwa 22620, Pakistan; javed.muhammad@uoh.edu.pk; 4 Department of Public Health/Nutrition, The University of Haripur, Khyber Pakhtunkhwa 22620, Pakistan; dramjadkhan77@uoh.edu.pk; 5 Medical Director of Informatics and Outcomes, St Joseph Mercy Oakland, Pontiac, MI 48341, USA; anupam.a.sule@stjoeshealth.org; 6 Department of Medicine Keystone Health, Penn State University School of Medicine, Hershey, PA 16801, USA; drraghutg@gmail.com; 7 Department of Medicine, Wellspan Chambersburg and Waynesboro (Pa.) Hospitals, Chambersburg, PA 16801, USA; 8 Research Center, Almoosa Specialist Hospital, Alahsa 36342, Saudi Arabia; abbas.almutair@almoosahospital.com.sa; 9 College of Nursing, Prince Nora University, Riyadh 11564, Saudi Arabia; 10 School of Nursing, Wollongong University, Wollongong, NSW 2522, Australia; 11 Administration of Pharmaceutical Care, Ministry of Health, Alahsa 31982, Saudi Arabia; saalhumaid@moh.gov.sa; 12 College of Medicine, Alfaisal University, Riyadh 11533, Saudi Arabia; awad.omari@drsulaimanalhabib.com; 13 Dr. Sulaiman Al-Habib Medical Group, Critical Care and Infection Control Department, Research Centre, Riyadh 11372, Saudi Arabia; 14 Department of Microbiology, Punjab Agricultural University, Ludhiana 141027, Punjab, India; dhawanmanish501@gmail.com; 15 The Trafford Group of Colleges, Manchester WA14 5PQ, UK; 16 Department of Veterinary Microbiology and Immunology, College of Veterinary Sciences, Uttar Pradesh; Pandit DeenDayal Upadhyaya PashuChikitsa Vigyan Vishwavidyalaya Evam Go AnusandhaSansthan (DUVASU), Mathura 281001, Uttar Pradesh, India; ruchi.vet@gmail.com; 17 Division of Surgery, ICAR-Indian Veterinary Research Institute, Mathura 281001, Uttar Pradesh, India; sharunkhansk@gmail.com; 18 Department of Chemistry, Government College of Engineering, Keonjhar 758002, Odisha, India; rkmohapatra@gcekjr.ac.in; 19 Department of Pharmacy, Faculty of Pharmacy, University of Dhaka, Dhaka 1000, Bangladesh; saikat-2018926336@pharmacy.du.ac.bd or; 20 School of Life Science and Food Engineering, Huaiyin Institute of Technology, Huaian 223003, China; bilaluaf@hyit.edu.cn; 21 Department of Mathematics and Statistics, Imam Mohammad Ibn Saud Islamic University, Riyadh 11432, Saudi Arabia; saalyami@imamu.edu.sa; 22 Department of Pharmacy, BGC Trust University Bangladesh, Chittagong 4381, Bangladesh; talhabmb@bgctub.ac.bd; 23 WHO Collaborating Centre on eHealth, UNSW Digital Health, School of Public Health and Community Medicine, Faculty of Medicine, UNSW Sydney, NSW 2052, Australia; 24 Division of Pathology, ICAR-Indian Veterinary Research Institute, Izatnagar, Bareilly 243122, Uttar Pradesh, India

**Keywords:** COVID-19, inflammatory cytokines, cytokine storm, immunopathology, genomics, immunomodulatory drugs

## Abstract

Coronavirus disease 2019 (COVID-19), caused by severe acute respiratory syndrome coronavirus 2 (SARS-CoV-2), is a severe pandemic of the current century. The vicious tentacles of the disease have been disseminated worldwide with unknown complications and repercussions. Advanced COVID-19 syndrome is characterized by the uncontrolled and elevated release of pro-inflammatory cytokines and suppressed immunity, leading to the cytokine storm. The uncontrolled and dysregulated secretion of inflammatory and pro-inflammatory cytokines is positively associated with the severity of the viral infection and mortality rate. The secretion of various pro-inflammatory cytokines such as TNF-α, IL-1, and IL-6 leads to a hyperinflammatory response by recruiting macrophages, T and B cells in the lung alveolar cells. Moreover, it has been hypothesized that immune cells such as macrophages recruit inflammatory monocytes in the alveolar cells and allow the production of large amounts of cytokines in the alveoli, leading to a hyperinflammatory response in severely ill patients with COVID-19. This cascade of events may lead to multiple organ failure, acute respiratory distress, or pneumonia. Although the disease has a higher survival rate than other chronic diseases, the incidence of complications in the geriatric population are considerably high, with more systemic complications. This review sheds light on the pivotal roles played by various inflammatory markers in COVID-19-related complications. Different molecular pathways, such as the activation of JAK and JAK/STAT signaling are crucial in the progression of cytokine storm; hence, various mechanisms, immunological pathways, and functions of cytokines and other inflammatory markers have been discussed. A thorough understanding of cytokines’ molecular pathways and their activation procedures will add more insight into understanding immunopathology and designing appropriate drugs, therapies, and control measures to counter COVID-19. Recently, anti-inflammatory drugs and several antiviral drugs have been reported as effective therapeutic drug candidates to control hypercytokinemia or cytokine storm. Hence, the present review also discussed prospective anti-inflammatory and relevant immunomodulatory drugs currently in various trial phases and their possible implications.

## 1. Introduction

The novel severe acute respiratory syndrome coronavirus 2 (SARS-CoV-2) is considered the cause of the ongoing coronavirus disease 2019 (COVID-19) pandemic. The pandemic has created huge challenges due to its accelerated dissemination and various clinical complications [1,2,3]. Presently, COVID-19 has affected more than 225 countries with nearly 140million confirmed cases [4,5] and 3 million deaths reported worldwide as of 15 April 2021 [4]. Recently, few potent vaccine candidates have been developed successfully, and the vaccination program is in progress at the global level to combat COVID-19 [6]; however, still effective drugs and therapeutics are awaited to be made available against this pandemic virus. The increase in the severity and outcome of SARS-CoV-2 infection in patients with pre-existing comorbidities [7,8]; the recent scenario of second wave of COVID-19 leading to a high increase in the number of cases [6]; emerging virus variants (SARS-CoV-2 UK variant B.1.1.7, South Africa B.1.351 variant and others) [6]; animal spillover events, cross-species jumping, and associated zoonotic concerns of this virus [9,10,11] have posed high challenges to the fight against COVID-19. It has been estimated that the virus takes about 5–14 days to incubate inside the human body [12]. The common clinical manifestations of the disease are reported as respiratory illness and high-grade fever. A few studies also reported other systemic manifestations such as neurologic symptoms and renal complications [3,13,14]. More reports on rare clinical conditions such as stroke-induced deaths and large vessel occlusions in the younger population are also reported. These studies indicate that the pathogenesis of the disease is more complex than thought. The exact reasons behind this predicament are still not available, although scientists have come up with two possible explanations, viz., (i) cytokine storm and (ii) disruption of the angiotensin-converting enzyme (ACE2) [15].

The pathogenesis of SARS-CoV-2 initiates when the viral particles invade the lung endothelial cells via attaching with the ACE2 receptors on cells’ surface, which in turn hyperactivates macrophages, natural killer (NK) cells, and other immune cells to release chemokines and cytokines [2,16]. This dysregulated and hyperinflammatory response eventually leads to inflated levels of the cytokines’ concentrations and can be considered the main plausible reason for multiple-organ damage [17]. Restraining the uncontrolled infiltration of cytokines is one of the effective ways to control COVID-19-induced multiple organ damage [18].

Though it is easy to identify the complications related to unregulated and increased levels of chemokines and cytokines, it is still difficult to distinguish between a normal and dysfunctional cytokines response as some cytokines are essential to generate an effective immune response to clear intracellular infections such as viral infection [19]. Hence, the present review intends to shed light on the molecular mechanisms of the cytokines and their functions and impact on the pathology of COVID-19. Furthermore, the immunomodulatory therapeutic strategies are also discussed to control and treat the cytokine storm or hypercytokinemia in critically ill patients with COVID-19.

## 2. Methodology of Literature Selection

A systematic literature review was performed to collect data by exploring authentic academic databases, including ScienceDirect, Scopus, PubMed, and Google Scholar, as well as public and government health organization websites, such as the World Health Organization (WHO), European Centre for Disease Prevention and Control of the European Union (ECDC), National Institutes of Health of United States (NIH), Guidelines International Network, and Chinese guidelines on Novel Coronavirus resources. For critical covering and compilation of most recent and relevant literature contents, the key terms searched include coronavirus, SARS-CoV-2, COVID-19, immunopathology, inflammatory cytokines, cytokine storm, immune functions, immunomodulatory drugs, and immunotherapies. After a literature assortment, the obtained data were carefully examined, and only closely matched studies were considered for critical discussion while excluding the irrelevant or generalized studies. Finally, 181 papers relevant to our review published up to 15 April 2021 were selected, and the review article was compiled and revised as per its title and theme.

## 3. Effect of COVID-19 Inflammatory Cytokines Response on Various Organs

SARS-CoV-2 elicits an innate immune response and causes an immediate rise in the neutrophils and other immune cells along with a marked reduction in the T cells (CD4+ and CD8+). However, the reduction of T cells along with the enhanced production of IL-6 and IL-8 has been reported as a remarkable characteristic of SARS-CoV-2 infection [20]. In patients with an inborn defect in IFN type I immunity and compromised immunity [21], inflammatory cytokines are produced in abundance, eliciting a cytokine storm also known as hypercytokinemia. This dysregulated and excessive cytokine production leads to pathological symptoms such as severe pneumonia, acute lung injury, and acute respiratory distress syndrome (ARDS) [22].

The systemic inflammatory response along with other co-morbid factors may lead to other diverse complications, such as cardiac failure, renal dysfunction, hepatic damage, and multiple organ disruption [2,23]; COVID-19 patients with a previous history of cardiovascular disease (CVD) show a very poor prognosis. A systemic analysis of COVID-19 effects reported that patients with a previous co-morbid history of high blood pressure (17%), hyperglycemia (8%), and heart disease (5%) are more prone to severe complications of COVID-19 compared to patients without any history of chronic medical conditions [24]. Another study reported that 7.2% of CVD patients infected with COVID-19 showed high troponin levels and electrocardiogram (ECG) anomalies, indicating potential cardiac damage [25].

## 4. Cytokinesand Chemokines Associated with Cytokine Storm

The word ‘Cytokine Storm’ was first used in an article on graft versus host tissue disease by Ferraraet al. in 1993 [26]. Since then, the term has drawn many scientific researches. The effect of the condition was much felt during the avian H5N1 influenza disease [27]. Numerous studies have suggested the significant roles of a range of pro-inflammatory factors such as interleukins (IL)-2, 6, 8, 10, 1β, tumor necrosis factors (TNF), interferons (IFN), and colony-stimulating factors (GM-CSF) in the prognosis of the COVID-19 disease. Additionally, chemokines, including CCL2, CCL3, CCL 5, and IP-143 10, have been reported as critical players in determining mortality during SARS-CoV infection. Moreover, the presumptive assumptions of the causative reason for multiple organ damage and mortality during SARS-CoV-2 infection have been closely linked to cytokine storm. Recently, Peng et al. (2021) also suggested that the cytokine storm plays a crucial role in the progression of SARS-CoV-2 infection and could be a major reason for multiple organ damage and an increased fatality rate in immunocompromised patients [28].

ILs, IFNs, TNFs, and colony-stimulating factors (CSFs) are the major cytokines involved in the generation of cytokine storms during COVID-19. Cytokines are broadly divided into two categories based on their functionality during the infection, such as pro-inflammatory cytokines/factors, including IL-6, IL-12, IL-1β, IFN, TNF, and anti-inflammatory cytokines/factors, viz., IL-4, IL-7, IL-10, and TGFβ [29,30,31].

The release of numerous cytokines is vividly associated with the emergence of multiple clinical manifestations, such as excessive IFN-γ secretion, resulting in headaches, chills, dizziness, fatigue, and fever. Like IFN-γ, TNF-α causes flu-like symptoms along with fever, fatigue, and malaise, but can also lead to lung damage, vascular leaking, heart failure, and synthesis of acute-phase protein [32]. The secretion of IL-6 causes vascular leak syndrome, triggering coagulation and complement pathways leading to the prominent indications of cytokine release syndrome, i.e., blockage of small blood vessels [33,34]. It is important to note that IL-6 is related to induce cardiomyopathies by the stimulation of coronary artery disease and myocardial dysfunction [35]. Moreover, severe cytokine release syndrome may also occur due to the activation of endothelial cells [32], and endothelial dysfunction can result in hypotension, capillary leakage, and impaired blood clotting. Overall, the outcomes suggest that the viral-triggered immunopathological responses might have a critical contribution to the progression of deadly pneumonia [25].

Cytokine storm is an extremely deadly immune disorder characterized by abrupt multiplication and hyperactivation of NK cells, macrophages, T cells, and the hypersecretion of over 150 chemical mediators and inflammatory cytokines by non-immune as well as immune cells [36,37]. The abnormal secretion of pro-inflammatory factors in viral invasions causes apoptosis of endothelial and epithelial cells of the lung, resulting in hypoxia, alveolar edema, and vascular leakage. Uncontrolled generation of chemokines (IP-10, CCL2, CCL3, and CCL-5) and pro-inflammatory factors (IL-1β, IL-6, IL-8, and GM-CSF) lead to scarring of the lungs and death [38].

Chien et al. [39] detected elevated serum concentrations of pro-inflammatory cytokines (IL-1, IL-12, IL-6, IFN-γ, and TGFβ) and chemokines (IL-8, CXCL10, CCL2, and CXCL9) in the case of SARS-CoV individuals. In contrast to moderate or mild disease, patients infected with severe form of MERS showed increased titer of pro-inflammatory cytokines (IFN-α and IL-6), and chemokines (CCL5, CXCL-10, and IL-8) [40]. A high level of proinflammatory CCR6, CCR4, Th17, and CD4 T cells by histological investigation of biopsy specimens from a deceased patient by SARS-CoV-2 infection suggests that hyperactivation of T cells might contribute to some extent to the serious immune damage [41]. Pulmonic investigation of patients with early SARS-CoV-2 also demonstrated irregular inflammatory cell penetration [42]. Taken together, the abnormal secretion of different cytokines leads to the activation of cytokine storm producing immunopathogenic injuries to various organs and tissues, even in the presence of a strong suppressing response of the immune system [43].

A network of cytokines and chemokines has been implicated in cytokine storm development in several recent studies. Furthermore, an array of chemokines, including CCL2, CCL3, CCL4, CCL7, CXCL1, CXCL3, CXCL8, and CXCL10, have been reported as critical players in the initiation and progression of the cytokine storm [44,45,46,47]. The analysis of bronchoalveolar lavage fluid (BALF) from severely ill patients with SARS-CoV-2 infection recently indicated that chemokines play a significant role in disease prognosis. Furthermore, in the peripheral blood mononuclear cells (PBMCs) isolated from the BALF of patients with COVID-19, overexpression of a set of genes encoding for several chemokines, including CCL2, CXCL10, CCL3, and CCL4, was recorded [47]. Similarly, in critically ill patients with SARS-CoV-2 infection, other recent studies have identified the overexpression of several genes encoding CCL2, CCL3, CCL4, and CCL7 in alveolar macrophages [47]. Furthermore, pulmonary observations from patients undergoing severe inflammation showed a substantial rise in specific immune cells, particularly inflammatory macrophages, along with the overexpression of several chemokines and cytokines, including CCL2, CCL3, CCL20, CXCL1, CXCL3, CXCL10, IL8, IL1B, and TNF [48]. Moreover, the fatality rate was found positively correlated with CXCL10 and CCL2 critically ill patients with COVID-19 [49].

Surprisingly, as opposed to patients with mild symptoms, chronically ill patients with SARS-CoV-2 infection had significantly higher transcription levels of chemokine-encoding genes. As a result, overexpression of CCL proteins or chemokines is linked to the incidence of SARS-CoV-2 infection [48]. Other studies have found higher levels of CXCL9, CXCL10, CCL2, and CCL3 in seriously ill patients relative to moderately infected patients, as well as higher levels of CXCL9, CXCL10, CCL2, TNFα, CCL7, and CCL3 in critically ill patients compared to moderately infected patients [46,50]. Moreover, clinical features of hyperinflammation, pulmonary damage, and respiratory dysfunction were positively correlated with the overexpression of chemokines and cytokines encoding genes in critically ill patients [48].

It is crucial to determine which chemokines are substantially relevant in the poor prognosis of the disease from a plethora of chemokines. In this sense, a recent study indicated that a collection of particular chemokines could play a pivotal role in recruiting specific immune cells such as monocytes. Furthermore, in some patients with COVID-19, the circulation of chemokines and immune cells may be positively associated with viral load. Furthermore, a substantial increase in CCL1, CCL2, CCL4, CCL8, CCL21, and CXCL9 was observed at the initiation of SARS-CoV-2 infection, which led to increased mortality risk in critically ill patients [51,52]. Furthermore, recent lung autopsy findings have suggested that seriously ill patients with COVID-19 have considerably higher levels of a set of chemokines, including CCL2, CXCL5, and CXCL6, than moderately ill patients [53]. As a result, several studies that corroborate each other strongly proposed that chemokines are crucially implicated in the poor prognosis, which results in a higher fatality rate [44,45,53,54,55].

Additionally, many studies have revealed that the release of cytokines happens only later in the respiratory cells and dendritic cells compared to the release of macrophages. The concentration of cytokines, TNF, and chemokines increase in the cells and surpasses the concentration of antiviral IFNs. Cytokine storm is likely to occur when excess inflammatory cells, such as monocytes or neutrophils, infiltrate into the tissues, resulting in cytokine-induced necrosis. This excessive production of cytokines followed by the infiltration of inflammatory cells plays a critical role in the disease progression [43,56,57,58,59].

This clinical impact can be better understood in SARS-CoV-infected animal models. The older animal primates suffered serious infection compared to the young primates, although the viral titers were nearly equal. The serious clinical impacts are more attributed to immune dysregulation than to viral infection. The possible reason for the fatality of the disease is more likely due to immune dysregulation than the viral infection itself [60]. Similarly, Rockx et al. [61] reported that disease severity in SARS-CoV-infected mice is closely related to disproportionate inflammatory gene signals. The disease is characterized by a rapid increase in inflammatory macrophages but a delayed IFN release, leading to rapid replication of the viral particles. The α/β-IFN receptors send specific signals to the influx of macrophages to emit chemoattractant that aid in more macrophage accumulation [62]. This pool of mononuclear macrophages produces large quantities of proinflammatory cytokines such as TNF, IL-6, and 1-β, leading to autoimmune disease severity. Furthermore, the inflammatory cytokines produced by the macrophages and IFNs substantially induce T-cell apoptosis, thereby increasing the viral infection [62]. Sometimes, the combination of excess-inflammatory cytokines and viral replication leads to entire tissue apoptosis, giving way to organ damage.

However, it a crucial to distinguish beneficial cytokines from harmful cytokines; beneficial cytokines such as IFN type-1 and IL-7 are essential to generate an appropriate immune response to clear viral infection. On the other hand, IL-1β, IL-6, and TNF-α are considered harmful cytokines and are involved in the generation of cytokine storms during the COVID-19 infection [31].

## 5. Cytokine Dynamics and Progression

The progression of cytokines plays a vital role in the immunopathogenesis of COVID-19. The entry of the viral antigens into the pulmonary epithelial cells elicits the first immune reaction, primarily by the innate immune modules of the immune system [62]. Apoptotic and necroptotic pathways are induced by the entry of viral particles into the lung epithelial cells, which in turn causes lung injury and the secretion of different chemokines. The first secretion of chemokines causes the recruitment of various immunologically important cells such as dendritic cells, plasmacytoid dendritic cells, neutrophils, and alveolar macrophages within the lungs. Toll-like receptors (TLRs) on alveolar macrophages recognize various molecular patterns such as viral particles, i.e., RNA, proteins, and other signals. This, in turn, activates the transcription and secretion of proinflammatory cytokines such as IL-6, IL-18 and IL-1β, and TNF-α. These pro-inflammatory cytokines further activate other immune cells such as B-cells, T-cells, NK cells, and other macrophages, which in turn secrete a large number of cytokines that lead to cytokine storms [31,63,64]. The defect in type-1 IFN immunity delays the effective immune response against SARS-CoV-2 [20]. This delayed response gives way to the uncontrolled multiplication of viral particles and undefined activation of interferons, attracting other immune modules such as neutrophils, monocytes, dendritic cells, and NK cells [52]. The abundance of these immune proteins attracts more cytokines, leading to the cytokine storm. The increasing viral load activates more cytokines, eventually causing hyperinflammatory reactions. This inflammatory pool of cytokines or hyper-activated immune cells infiltrates into the microvasculature and alveolar epithelium, leading to severe vascular emanation and edema giving way to ARDS. In severe conditions, the inflammatory reaction disseminates to other parts, leading to multi-organ damage [65].

## 6. Molecular Mechanisms Linking Inflammatory Cytokine in Diseases

An increase in cytokine production is associated with a high degree of pyrexia, blood leakage, the formation of multiple blood clots, and pleural effusion. Acute Respiratory Distress Syndrome (ARDS) is a common adverse effect of COVID-19 based on excessive cytokine production [66,67]. During an episode of ARDS, the titer values of proinflammatory cytokines and chemokines are soared up, along with high viral titers. High concentrations of interleukins (IL-1B), IFNs (IFN-γ), monocyte chemoattractant protein (MCP-1), and IFN-induced proteins (IP-10) have been identified in patients with COVID-19 [68]. The release of these specific cytokines is likely to activate the T-helper-1 (Th-1) cells, which elicit specific immunity [68,69]. During SARS-CoV-2 infection, these specific cytokines also activate T-helper-2 cell cytokines (IL4 and 10) that cause a negative inflammatory response, leading to more disease severity [70]. In comparison to patients with COVID-19 in general wards, intensive care unit (ICU) patients showed high titers of serum MCP-1, IP-10, TNF-*α*, macrophage inflammatory protein-1A, and granulocyte colony-activating factor. The outcomes of these studies might reveal the positive correlation of cytokine storm with disease severity. In a retrospective study, Yang and coworkers (2020b) reported that 71.2% of patients (37) necessitate mechanical ventilation, whereas 67.3% of patients (35) suffered from ARDS in case of severe SARS-CoV-2-infected pneumonia. Furthermore, ARDS results in a significantly enhanced mortality rate in older patients. The key pathological alterations in ARDS are injuries of interstitial and pulmonary tissues due to infiltration of the nonspecific inflammatory cells [71,72]. The local release of excessive cytokines is the pivotal factor inducing clinical symptoms and pathological alterations [73]. A high expression of cytokines level in individuals with ARDS is certainly associated with an increased death rate [74].

Previous studies in related diseases such as SARS and MERS reveal that the treatment in severe cases largely depends on decreasing the viral load and inflammatory marker concentration. Cytokine storm is a concoction of many inflammatory elements such as colony-stimulating factors, IFNs, ILs, chemokines, and TNF-α (Table 1).

ACE2 is a well-known cell receptor for SARS CoV-2 [86,87]. Moreover, 21 mutations have been identified in the binding region of S glycoprotein, suggesting coronavirus has gradual variations to acclimatize human hosts [88,89]. Though ACE2 is present in different tissues, including renal tubular epithelium, vascular endothelial cells, and coronary arteries [90,91], the virus mainly attacks pulmonary alveoli and macrophages [92], explaining the main reason for acute lung damage. Generally, all populations are vulnerable to SARS-CoV-2. In animal studies, older rhesus monkeys are observed to be highly prone to COVID-19 [60], and ACE2 overexpression in the lower respiratory system might enhance susceptibility to the virus [93,94]. It was determined using genetic analysis that ACE2 expression was higher in East Asians, and results may demonstrate differential SARS-CoV-2 infection extent in distinct populations [95]. Additionally, fast replication of the virus leads to immune evasion, cell pyroptosis, and anti-Fc antibodies-induced lysis of the cell. All these factors cause the secretion of a large number of chemokines and pro-inflammatory cytokines. Thus, the clinical manifestations of COVID-19 patients are attributed to combined cytopathogenic consequences and immunopathological damages triggered by intense cytokine storms [30]. Recently, four different molecular pathways were suggested to induce the overproduction of inflammatory cytokines: (a) the downregulation of ACE2 through upregulation of the renin-angiotensin-aldosterone system (ACE/angiotensin II/AT1R), (b) mas receptor attenuation (ACE2/MasR), (c) hyperactivation of bradykinin, and (d) activation of the complement system through C5a and C5b-9 proteins [30]. Furthermore, there is an urgent need to elucidate a clear portrait of molecular pathways involved in the generation of cytokine storm and this will allow exploring the effective therapeutic strategies to treat ARDS in severely ill patients [96].

## 7. Cytokine Profile of COVID-19 Patients with and without Organ Disorder

Reports suggest that COVID-19 may increase the concentration of liver enzymes such as bilirubin and aminotransferase that are indicative of hepatitis and hepatic damage. Explicit studies on COVID-19-induced liver damage are deficit. Some other factors such as myositis also steer the rise of liver parameters when coupled with COVID-19 [97].

In the lungs, the αβ and γ IFNs permeate into the cell using two pathways, viz., the FasL pathway and the DR5 pathway. The death of endothelial and epithelial cells leads to the breakdown of microvascular and alveolar epithelial cell blockades, facilitating vascular leakage and hypoxia. In patients with COVID-19, the severity of the disease is directly proportional to the concentration of IL-2R and IL-6, distinguishing the disease status by critically ill, severely ill, and normal patients [70]. However, pre-existing cardiovascular problems in some patients are linked to higher levels of ACE2, which may lead to a stronger inflammatory response against SARS-CoV-2 infection and lead to severe cardiac problems in some patients [7,98,99]. Furthermore, the latest clinical study suggested that multiple organ damage and immune dysregulation are the characteristic features of severe illness, and these features can be linked with the hyperinflammatory response and variable expression of ACE2 among critically ill COVID-19 patients [100].

## 8. Immunology and Genomics of Cytokines Storm

In special conditions such as secondary infections, the cytokines take up several unconnected tasks depending on the target antigen and availability of other cytokines. Depending on the necessity, these cytokines can transduce the signals through intracellular mechanisms. The cytokines are a collection of complicated network modules with interrelated and redundant genetic pathways [101]. These unusual features of cytokines make the evaluation of therapeutic utility more difficult.

In the cytokine storm, the IFNs play a critical role [75]. There are different classes of IFNs as given below:

- Type I IFNs- IFN α and IFN β. They signal through a heterodimeric receptor complex called IFN AR1 or AR2;
- Type II IFNs—IFNγ. They signal through the IFN-γR1/IFN-γR2 complex;
- Type III IFNs—IFNλ1, IFNλ2, IFNλ3. These types of IFNs are correspondingly denoted as IL -28A, IL-28B, and IL-29. They signal through the JAK-STAT pathway.

The ILs are diverse types of immune modulators that assist in the proliferation and differentiation of immune cells. Their functions are assorted, including the activation of pro-inflammatory and inflammatory responses. Other significant functions include eliciting primary-phase signals, activating epithelial immune reactions, and acting as transport molecules to immune cells to the infection site. More functions of ILs are yet to be explored. The origin, functions, and designation are often confused with interferons because some ILs share identical characteristics and functions to those of IFNs [102].

Various signaling pathways such as IFN regulatory factor-3 (IRF3), nuclear factor κB (NF-κB) pathway, Janus kinase (JAK)/signal transducer, and activator of transcription (STAT) signaling pathways have been reported in the antiviral response and the generation of the cytokine storm. These pathways are usually activated by TLRs [103,104]. Different chemokines belong to a massive family of more than 44 immune proteins. These cytokines are multi-targeted and are specific to more than 21 G-protein receptors. These immune proteins are categorized as CXC, CC, C, and CX_3_C based on their cysteine positions. They regulate the immune cell transportation and assist in many-body mechanisms such as embryogenesis, immune system development, and other instances such as cancer metastasis. They are more involved in the proinflammatory process and elicit diverse immune cells—including neutrophils, macrophages, and lymphocytes—during infections [105]. Colony-stimulating factors (CSF) assist in the cascade inflammatory reaction during any infection. The three major classes of the CSF—namely granulocyte-colony CSF, granulocyte-macrophage CSF, and macrophage colony CSF—spur the multiplication and differentiation of hematopoietic progenitor cells [78]. Tumor necrosis factors (TNFs) are the most explored proinflammatory cytokines that are released to suppress tumor growth. The functionality of the immune protein is best seen in the pathogenesis of severe viral, bacterial, and parasitic diseases and cancer. Abnormal production of the proteins is a major cause of autoimmune disorders and other inflammatory diseases [106].

## 9. Cytokine Levels during COVID-19

Clinical manifestations during SARS-CoV-2 infection usually predict the activation of different cytokines. Pyrexia, malaise, and chills may predict an increase in the interferon levels, while acute symptoms, such as vascular damage and lung contusions, along with general symptoms may indicate high TNF levels [107]. A high concentration of interleukins may lead to diffused intravascular coagulation (DIC), a characteristic phenomenon of extreme cytokine release syndrome (CRS) [35]. The IL-6 immune protein solely can lead to cardiomyopathy during serious CRS conditions. A high concentration of activated endothelial cells may lead to capillary damage, low blood pressure, and coagulopathy. These events singularly or together effectuate serious repercussions or even mortality [32]. A recent study on the inflammatory biomarkers deduces that the geriatric population with high IL-6 baseline levels and high increment scale of overtime IL-6 levels are more prone to multiple systemic diseases than with low overtime increment of IL-6 with high baseline IL-6 titers. The soaring IL-6 levels during SARS-CoV-2 infection activate CRP, another inflammatory culprit to COVID-19-based multiple systemic disorders and pneumonia. Huang et al. (2020) [19] studied clinical manifestations of SARS-CoV-2 infected patients in Wuhan, China and concluded that the prognosis is bad for patients with COVID-19 with a higher concentration of inflammatory markers and symptoms of ARDS. Another similar study conducted in the same locality reported that serious patients with COVID-19 showed high titers of neutrophils and low concentration of lymphocytes, eventually leading to cytokine storm. It could be deciphered that inverse proportionality in neutrophil versus lymphocyte count indicates acute systemic inflammation [108]. This neutrophil-lymphocyte ratio can serve as a prognostic marker in extreme cases. Similar findings have been reported by Wang et al. [25], which can confirm the credibility of the previous findings. Another study reported that a combination of factors—viz., high concentration of IL-6, IL-2, IL-10 and TNF-α, low concentration of CD4^+^ and CD8^+^ T cells, and lymphopenia—are characteristic of severe systemic infection [70]. The platelet to lymphocyte ratio also is associated with disease severity [109]. Another valuable prognostic marker is the serum viral load versus IL-6 levels [70]. Studies have specified that excessive concentration of IL-6 indicates more disease severity. Gao et al. (2020) [110] reported that the IL-6 and D-Dimer ratio are good early prediction markers for pneumonia and systemic disease development. Early correlation of these markers can play a significant role in the treatment of COVID-19 disease.

## 10. Overview on Multisystem Organ Failure and Death Due to COVID-19

The major reason for multiple organ failure and mortality due to COVID-19 is the excessive and uncontrolled immune system response. A frail immune system can increase viral titers, but generally does not cause multi-organ failure. A hyperimmune response is the leading cause of multi-organ failure and death through excessive inflammatory proteins or hemophagocytic lymphohistiocytosis. Cytokine storms can trigger other secondary conditions such as hyperferritinemia, incessant high-grade pyrexia, and cytopenia, which together can lead to serious debility or death [111]. Ruan et al. [112] stated that the patients who succumbed to COVID-19 had higher titer values of IL-6 and ferritin in comparison to convalesced COVID-19 patients. This report is in accordance with other similar studies, suggesting that a pattern indicating high values of neutrophil-lymphocyte ratio and inflammatory biomarkers with low titers of basophils, eosinophils, monocytes, NK cells, B-cells, suppressor cells, and helper T-cells are predictive of serious repercussions, including multi-organ failure and death [72,108]. Other researchers have also indicated septic shock in 4–8% of patients with severe disease [113].Moreover, eosinopenia has been associated in up to 81% of cases and was proposed as a possible diagnostic marker for the disease. Persistent eosinopenia was associated with higher mortality [114]. Using flow cytometry, Xu et al. [41] found a significant reduction in the numbers of peripheral CD8^+^ and CD4^+^ T cells, whereas both these cells were hyperactivated. High concentrations of cytotoxic granules, such as granulysin and perforin, were observed in CD8^+^T cells. In addition, the level of proinflammatory Th17 cells was also found to be increased. It can be inferred that over-activation of pro-inflammatory Th17 and CD8^+^T cells is likely to contribute to serious immune impairment. After the SARS-CoV-2 exposure, CD4^+^T lymphocytes were quickly stimulated to become hyperactivated Th1 cells, along with the detection of inflammatory CD14^+^and CD16^+^monocytes in peripheral blood [115]. In another report, NK cells were 34.31% and 47.62% reduced in the case of slight and severe COVID-19, respectively [18]. Some other patients with COVID-19 revealed typical diffuse alveolar injury along with infiltration of macrophages and monocytes instead of lymphocytes, demonstrating the role of macrophages in a cytokine storm [116,117].

## 11. Immunopathology and Role of Systemic Cytokines in Inducing Emergency Myelopoiesis during SARS-CoV-2 Infection

Severe COVID-19 manifestations are often accompanied by secondary bacterial infection and sepsis. Bacterial sepsis is always a bother given the challenges of patient heterogeneity [118]. To date, the vast complications of bacterial sepsis are not raveled. Filbin et al. [119] reported that the plasma samples from patients with COVID-19 with extreme bacterial sepsis activate emergency myelopoiesis and expression of CD^14+^monocyte state [119]. The CD^14+^ monocytes from these patients show low stimuli response and express human leukocyte antigen (HLA) rather marginally. These monocyte cells also repress the functionality of the immune compartment such as monocytic myeloid-derived suppressor cells (MMDs) [120].

The monocyte small conditional RNA sequence data (scRNA-seq) from sepsis COVID-19 patients revealed a gene expression pattern of MS1 marker genes ALOX5AP, *IL1R2,* and *RETN* among other 20 genes [121]. Co-expression probing revealed a high genetic correlation with *S100A8* genes. This similarity indicates that the gene sequence plays a major role in MS1 gene expression [122]. Previously, the gene sequence was studied to play a part in the development of MMDs in several cancers and sepsis. Transcriptional data studies report the relationship between MS1 gene expression and severe sepsis [123]. It has also been found that the MS1 gene dataset is inversely proportional to patient survival, while the MHC-II gene set is directly proportional to survival. This finding is a remarkable prognosis factor for COVID-19-induced sepsis.

The pattern of MS1 cell expansion during COVID-19 infection was studied in four COVID-19 scRNA-seq datasets. The results indicate that the MS1 expression is increased in extreme bacterial and viral infections, indicating the prognostic value of MS1 cells during secondary infections [124,125,126]. Lee et al. deduced that the cytokines in patients with COVID-19 trigger the differentiation of MS-1 cells from the hematopoietic stem cells [127]. Their outcomes also report that plasma from potential sepsis COVID-19 patients greatly stimulates the formation of neutrophils and monocytes compared to healthy adults. Additionally, the HSPCs activate the CD14+ cell production with considerable levels of MS1 on incubation.

**Figure 1:**
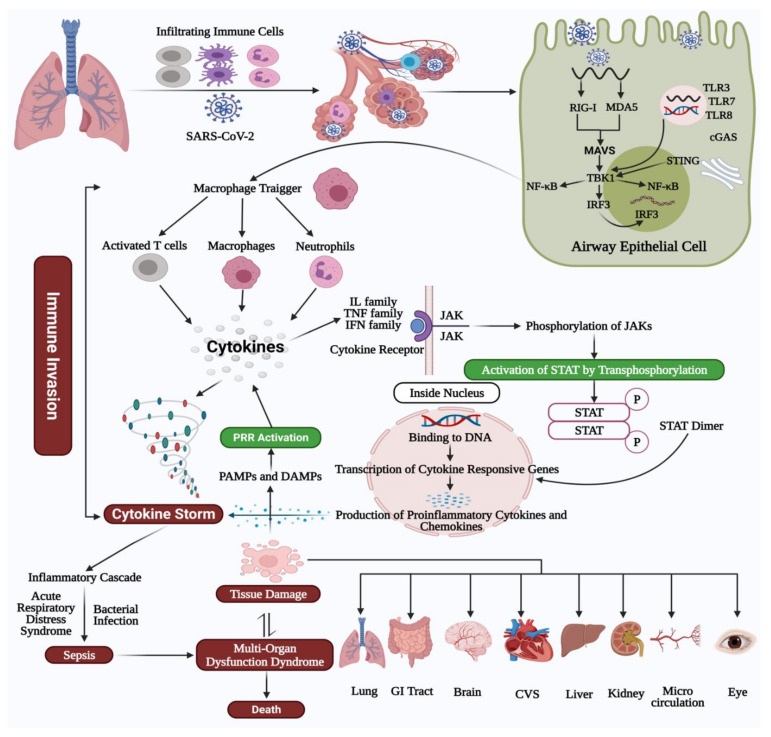
Molecular mechanisms, immune functions, and immunopathology of inflammatory cytokines in COVID-19. Overview of innate immune sensing annotated with the known means by which SARS-CoV-2. Following entry in the lung airway epithelial cell, the virus RNA genome may be recognized by pattern recognition receptors (PRR)s, including endosomal toll-like receptor (TLR)3, TLR7, TLR8 recognizing double-stranded and single-stranded RNA. The virus may be recognized in the cytosol by the retinoic acid-inducible receptor (RIG)-I or the melanoma differentiation-associated protein (MDA)5. Following viral recognition by PRRs, this triggers signaling through IFN regulatory factor (IRF)3 and NF-κB to induce IFNs and pro-inflammatory cytokines. NF-κB can also activate the macrophages, which contribute to increased cytokine levels and the cytokine storm. Cytokine binds to a specific receptor and allows transactivation of the associated Janus Kinases (JAKs). Activated JAKs then phosphorylate tyrosine on the receptor’s intracellular domains, which recruit the Signal Transducers and Activators of Transcription (STAT) transcription factors. STATs are translocated into the nucleus and upregulate the transcription of cytokine-responsive genes. This essentially causes immune invasion, leading to clinically relevant conditions such as acute respiratory distress syndrome (ARDS), sepsis, MODS, and potentially even death. The organs affected due to multiorgan dysfunction syndrome (MODS) and their associated symptoms have been shown. Abbreviations: SARS-CoV-2, severe acute respiratory syndrome coronavirus 2; COVID-19, coronavirus disease-2019; RIG-I, retinoic acid-inducible receptor; MDA-5, melanoma differentiation-associated protein; MAVS, mitochondrial antiviral-signaling protein; TBK1, TANK-binding kinase-1; IRF3, interferon regulatory factor; NF-κB, nuclear factor kappa-light-chain-enhancer of activated B cells; STING, stimulator of interferon genes; TLR, toll-like receptor, cGAS, cyclic GMP–AMP synthase, PRR, pattern recognition receptors; PAMPs, pathogen-associated molecular patterns; DAMPs, damage-associated molecular patterns; IL, interleukin; TNF, tumor necrosis factor; IFN, interferon; JAK, Janus Kinase, STAT, signal transducer and activator of transcription; ARDS, acute respiratory distress syndrome; MODS, multiorgan dysfunction syndrome.

An overview of molecular mechanisms, immune functions, and immunopathology of inflammatory cytokines in COVID-19 is depicted in Figure 1.

## 12. Regulation of Pro- and Anti-Inflammatory Cytokines via Immunomodulatory Drugs and Immunotherapeutics

The most sagacious treatment method to control the serious effects of COVID-19 is to repress the anti-inflammatory and pro-inflammatory cytokines before these progress into a cytokine storm. Various immunomodulators and immunosuppressors as therapeutic drug candidates have been suggested to control the cytokine storm [128]. In this context, several immunomodulatory therapeutic options are being used to reduce the elevated cytokines, which activate a hyperinflammatory response among severely-infected cases with SARS-CoV-2 infection [129]. A plethora of therapeutics have been suggested and used to control the hyperinflammatory response, including corticosteroids (such as dexamethasone), antiviral drugs, hydroxychloroquine, and interleukins antagonists such as tocilizumab, sarilumab, and anakinra [93,130,131,132]. Additionally, inhibitors of essential signaling pathways such as TNF-α inhibitors and Janus kinase inhibitors have also been suggested as possible treatment forms to treat severe SARS-CoV-2 infections [133,134,135].

Among all these therapeutic drugs, corticosteroids are used widely to control the excessive release of cytokines in autoimmune diseases and cancers. These drugs, when administered timely, will aid in curbing the systemic inflammatory syndrome and ARDS. Certain practical impediments such as impaired viral clearance and secondary infections also need to be considered when contemplating to administer corticosteroids in the early stages of the disease. However, some studies conducted on patients with pneumonia showed a higher fatality rate and increased secondary infections in patients administered with corticosteroids [122]. Hence, it has been suggested that corticosteroids might not be recommended for cytokine regulation in patients with COVID-19. Conversely, several other studies suggested that corticosteroids such as glucocorticoids and mineralocorticoids are effective immunomodulatory candidates in controlling the cytokine storm in critically ill patients with COVID-19. Corticosteroids such as dexamethasone and methylprednisolone have been considered effective therapeutic agents to control the pro-inflammatory cytokine secretions, such as TNF-α, IL-1, and IL-6. The use of corticosteroids may be an efficient strategy to treat acute COVID-19 patients, particularly those who require mechanical ventilators [104,131,136]. Interferons form an important component of host antiviral immunity. The use of glucocorticoids/corticosteroids such as dexamethasone and methylprednisolone can be directly suppressed the activity of interferons and may affect the ability of host to counter the virus. Therefore, a retrospective study was conducted to investigate the potential of therapeutic interferons to compensate the loss of antiviral immunity induced by glucocorticoids [137]. The findings from the study indicate possible synergy when interferons and glucocorticoids are used in combination for managing COVID-19.

Anti-malarial drugs such as chloroquine and hydroxychloroquine were prospective drug candidates for patients with COVID-19 as they regulate the production of cytokines, interfere in receptor-signaling pathways, and restrict viral entry. During the early stages of the pandemic, the drug was prescribed as the main drug with or without macrolides [40]. However, recent studies have reported more mortality rates induced by ventricular arrhythmias in patients who were administered the drug. Following the results, the WHO has laid severe restrictions on the common administration of chloroquine/hydroxychloroquine to patients with severe COVID-19 disease.

Convalescent plasma therapy, antibodies, intravenous immunoglobulins, and monoclonal antibodies-based therapies also constitute potent immunomodulatory therapeutic options being used during COVID-19 to treat severely ill patients that aid in reducing the associated mortality rate [138,139,140,141]. Oral polyvalent immunoglobulins (PVIG, KMP01D) have also been suggested to modulate and/or block cytokine storm effectively and safely [142]. Mesenchymal stem cell (MSCs) therapy is also being explored for its potential in treating and managing patients with COVID-19. Possessing strong immunomodulatory and regenerative activities, MSCs are considered promising candidates to treat or ameliorate the severity of SARS-CoV-2 in patients with COVID-19, lessen ARDS and halt cytokine storm, improve pulmonary function, and reduce morbidity and mortality. Recent clinical trials proved these to be safe and efficacious in reducing overall outcome of the disease, though few limitations and challenges need to be addressed for their full practical utility during pandemic as well to have high value as a next-generation therapy and to counter future challenges [141,143,144,145,146,147].

Monoclonal antibodies such as tocilizumab (TCZ) and sarilumab both compete with IL-6 for IL-6 receptor sites and effectively regulate cytokine storm in severe cases and have been used in several clinical trials to treat patients with COVID-19. TCZ, a recombinant monoclonal antibody (MAb) against IL-6 receptor, has been used to reduce inflammation and to treat rheumatoidarthritis [148]. TCZ is also approved for cytokine release syndrome treatment. Pro-inflammatory cytokines such as IL-6 (a crucial cytokine), along with C-reactive protein and ferritin, are involved in the mediation of fever and the acute inflammatory response. TCZ has also been used in the treatment of COVID-19 in a number of critically ill hospitalized patients. Tocilizumab is being used in patients with serious COVID-19 manifestations, even those with serious disease. In China, a recent research experiment reported on the outcomes of patients treated with TCZ. Tocilizumab @400 mg (Roche Pharma Ltd., Schweiz/Roche Holding AG, Basel, Switzerland) was given subcutaneously to 21 patients who were suffering from severe COVID-19 pneumonia in this uncontrolled research. The fever has been observed again in treated patients within a few days; however, 15 out of 20 had their oxygen requirements reduced, and one patient did not require any additional oxygen treatment. Computerized tomography (CT) scans of the chest improved in 19 of the 20 participants [149].

JAK-STAT receptor inhibitors such as baricitinib have been reported to prevent viral invasion into the host cells and control the hyperinflammatory reactions in COVID-19 therapeutic interventions [150,151]. Inhibition of the JAK pathway represses the signal transduction initiated by the binding to the IL-6 receptor. Then, the use of immunosuppressors to inhibit the JAK/STAT pathway is also considered as an effective therapeutic method to control the hyperinflammatory response [21,152]. Additionally, it was suggested that JAK antagonists or inhibitors may not always be important in the earlier phases of SARS-CoV-2 infections because of the action of interferons, which has been a significant mediator of viral elimination in the body facilitated through the JAK-STAT signaling pathway. Hence, the signaling pathway (JAK-STAT) inhibitors were suggested as a therapy for severe SARS-CoV-2 infections with similar characteristics to the cytokine storm [150,153]. Furthermore, in COVID-19 therapy, ruxolitinib (Henan DaKen Chemical Co., Ltd., Zhengzhou, China), a JAK-STAT antagonist that inhibits IFN-gamma, is being used [154,155], and TNF-suppressing antibodies such as adalimumab and golimumab were also employed to manage COVID-19 cases with severe immune response [156].

In conjunction, a broad range of TNF inhibitor formulations are currently being used, particularly humanized TNF-α-inhibitors, namely adalimumab and infliximab, to treat COVID-19 and ameliorate cytokine storm [130,157]. TNF-α has been shown to be a main cytokine that is released in a variety of ailments that lead to inflammation, including in the acute and chronic phases, as evidenced in its effectiveness as a therapy for a wide spectrum of ailments [158]. Blocking TNF-α causes a decline in IL-1 and IL-6, as well as adhesion molecules and vascular endothelial growth factors (VEGFs), and in disorders such as autoimmune diseases, TNF blockers are being suggested to be used in hospitalized patients with severe inflammatory response [159].

Apart from the conventional therapeutic candidates, various molecular therapeutic strategies are being pursued to counter the cytokine, storm such as targeting the microRNAs which are essential to produce cytokines. MicroRNAs and microRNA network targeting might be a useful technology to develop faster and effective therapeutic approaches to treat hyperinflammatory responses generated during SARS-CoV-2 infection [160]. In addition to the use of anti-inflammatory therapeutics to treat pneumonia associated with COVID-19, immunostimulant drugs offer additional advantages to manage recurrent bacterial superinfections that can occur in the later course of the disease [161]. Lethal forms of COVID-19 can be attributed to the insufficient and/or inefficient immune responses associated with the hyperinflammatory syndrome/cytokine storm. Therefore, therapeutic approaches for managing COVID-19 should suppress the extreme inflammatory responses; at the same time, they should maintain the normal immune system and its response against SARS-CoV-2 [157,162,163].

Besides that, multiple different phytochemicals found in pharmaceutically important plants may seem to have established immunomodulatory ability in regulating and controlling the hyper-inflammatory reactions, and therefore are currently being investigated for potential therapeutic and preventive capacity in patients with COVID-19 [6,9,164,165,166]. Traditional Chinese medicine (TCM), a medical system that has continued to develop over the past decades, also offers several preparations that can be used for managing COVID-19. TCM herbal medicine contains several herbs and the combinations have significant bioactivity and therapeutic potential [167,168]. The beneficial effects associated with the use of TCM can be attributed to their anti-inflammatory and immunomodulatory activity [168]. In a retrospective cohort study conducted among 300 patients with COVID-19, the combined use of Western medicines and TCM was found to be associated with shorter hospital stays due to faster recovery [169]. Lianhuaqingwen (LH) is a TCM formula that possesses broad-spectrum antiviral and immune regulatory activity against respiratory viruses and has been previously used to treat influenza [29]. Recently, LH was found to inhibit SARS-CoV-2 replication and reduce the production of pro-inflammatory cytokines such as TNF-α, IL-6, and monocyte chemoattractant protein 1 in Vero E6 cells [167].

An overview on the prospective immunomodulatory drugs and therapeutics to control extensive cytokine production in patients with COVID-19 is presented in Table 2.

## 13. Conclusion and Future Perspectives

COVID-19 is an ongoing pandemic and has caused a considerable number of deaths around the world. The severity of the disease and mortality rate has been linked to the elevated secretion of inflammatory cytokines. The hyperinflammatory response in severe COVID-19-infected patients leads to pneumonia, lung failure, and multiple organ damage. However, inflammation is considered one of the protective gears of the immune system as many potential pathogens are initially identified and eradicated through the body’s defense mechanism. Diverse units of the immune system work in conjunction to elicit an immune response. Inflammation is one such response where many immune modules work together. However, the response can become a precursor of trouble with uncontrolled immune protein activation leading to indefinite systemic complications. In SARS-CoV-2 infection, there is a thin line between the effectiveness of cytokines and their harmfulness. Overexpression of cytokines such as interferons, interleukins, and TNF-α can lead to cytokine storms, whereas expression of some ILs and INFs is essential to generate an immune response to clear viral infections. Several recent studies linked the higher mortality rate in hospitalized patients with COVID-19 with the over-expression of a plethora of inflammatory cytokines. Hence, it can be suggested that to reduce the mortality rates among the severely ill COVID-19 patients, effective and earlier regulation of the cytokine storm using treatments including immune suppressants, immune modulators, and cytokine inhibitors is imperative. Moreover, harnessing the expression of certain specific cytokines (including the proinflammatory and anti-inflammatory cytokines) will lead us to understand the prognosis of the disease and help in the development of better therapeutic approaches in the future.

Presently, a variety of therapeutic drug candidates with various forms of modes of action have been considered to combat extreme hyperinflammatory responses in hospitalized COVID-19 patients. From a tsunami of upcoming clinical research studies, it can be concluded that cytokine antagonists such as Tocilizumaband Sarilumab (Regeneron Pharmaceuticals, Inc., New York, USA), and JAK-STAT inhibitors such as Ruxolitinib and Baricitinib (AnHuiHaiKang Pharmaceutical Co., Ltd., Anhui, China), represent potential candidates in managing severe inflammatory response or cytokine storm. Convalescent plasma therapy, antibody-based therapies, mesenchymal stem cell therapies, and medicinal herbs have shown potential to combat the disease severity, lessen cytokine storm, and reduce mortality in severely ill patients with COVID-19. Nevertheless, further research is needed in the near future to fully comprehend the molecular mechanisms of such prospective drugs for treating SARS-CoV-2 infection in critically ill patients. Additionally, more strategies to control the unregulated expression of inflammatory markers/cytokines are in the pipeline. However, it is also an urgent requirement to distinguish the inflammatory and anti-inflammatory response to develop better therapeutic drug candidates.

## Figures and Tables

**Table 1:** Significance and functions of inflammatory factors involved in the cytokine storm.

Sl. No.	Inflammatory Factors	Important Candidates	Family	Function
1.	Interferons	IFN-γ	Cytokine family	Elicits innate immunity Directs expression of the antiviral protein by expressing specific coding genes [75,76]
2.	Tumor Necrosis Factor	TNF-α	Cytokine family	It is a pyrogen released during serious infection Significant marker for chronic inflammatory and autoimmune diseases [77]
3.	Colony-Stimulating Factor	G-CSF, M-CSF, and GM-CSF	Hematopoietic growth factors	Activate the different signaling pathways that produce macrophages and granulocytes [78] Helps sustain the inflammatory reaction
4.	Interleukins	IL-6, IL-8, IL-9, IL-10, IL-1b, IL-17, and IL-18	Cytokine family	Assist in cell differentiation and activation Act as transporters of immune cells to the infection site [79] Invigorates acute phase signaling and triggers epithelial cells Signal the production of secondary cytokines Help in activation of T-helper 17 cells Marker for disease severity [70,80,81]
5.	Chemokines	IP-10, MCP-1, MIP 1-α	Cytokine family	Major chemo-attractants that aid in the transportation of immune cells [82] Regulate cell growth and differentiation and control immune response [83,84] Regulate tumor growth and metastasis [85] Serve as a significant marker to test the severity of ARDS

**Table 2:** Potential immunotherapeutic drug candidates to treat COVID-19 patients.

Sl. No.	Drug and Treatment Approach	Drug Candidates	Mechanism of Action	Limitations
1.	TNF-α Blockers [157,170]	Adalimumab, Atanercept, and Infliximab (Remicade)	Block excess activation of inflammatory cytokines by blocking the TNFR1 receptor and controlling the TNF-dependent cytokine cascade	Blocking the TNFR1 receptor can lead to a less efficient immune system
2.	C-C Chemokine Receptor Type 5 Inhibition [171,172]	Leronlimab, Maraviroc, Vicroviroc, and Aplaviroc	Inhibit the C-C Chemokine receptor which is expressed on the surface of immune proteins to facilitate release of cytokines	The safety and efficacy of the drug are yet to be confirmed. A clinical study is currently underway (NCT04347239)
3.	Janus Kinase Inhibitors [150,154,155,157]	Ruxolitinib, Baricitinib, Jakotinib, Ruxolitinib, Tofacitinib, Perficitinib, Filgotinib, Fedratinib, Ipadacatinib, and Fostaminib	Foster endocytosis of viral particles and repress cytokine production	Largely suppress cytokine production especially interferons leading to compromised immune response
4.	Cytokine Targeting Therapy or Cytokine inhibitors [32,134,149]	Tocilizumab, and Sarilumab	Many receptor blockades and antagonists like IL-1 blockade drugs, IL-6 antagonists assist in targeting the interleukin activation	The clinical trial results are encouraging and drugs like Tocilizumab are administered in patients with end-stage refractory hypoxemia
5.	Interferon Therapy [173]	Interferon-lambda (IFN-λ) and interferon gamma (IFN-γ)	Possess broad-spectrum antiviral (slows viral replication and dissemination) as well as immunomodulatory properties	Can facilitate bacterial superinfection by reducing neutrophil recruitment. The adverse effects can be minimized by reducing the duration of treatment
6.	Mesenchymal Stem Cell Therapy [144,145,174,175,176,177]	N.A. *	Regulates immune response as immunomodulator, reduces inflammation, act as regenerative medicine and halt cytokine storm in severely ill COVID-19 patients	Clinical trials have proven the safety and efficacy of this treatment modality, though a few limitations and challenges need to be addressed
7.	Intravenous Immunoglobulin (IVIG) Therapy [132,139,178,179]	N.A.*	Passive immunoglobulin therapy helps in viral clearance without activating systemic cytokine cascade attacks	Although the treatment is effective, no efficacious results were found in patients with advanced lung injury or systemic inflammatory reactions
8.	Convalescent Plasma Therapy [132,138,139,180]	N.A.*	Elicits immunomodulatory effects and clears viral load	The efficacy and safety of the treatment is comparatively higher than all other interventions. More studies are in the pipeline
9.	Corticosteroids [131,143,181,182]	Dexamethasone, Methylprednisolone, and Prednisone	Inhibit pro-inflammatory cytokines including IL-6 and IL-8. Inhibition of IFN-gamma expression in Th1 immune response along with downregulation of IL-4 gene expression in the Th2 immune response	Several side effects are associated with corticosteroids use such as skin problems, electrolyte disturbances, high blood pressure, high blood sugar, pancreatitis, immunologic, neurologic, and neuropsychological effects
10.	Herbal Therapy [23]	Withanolides (*Withaniasomnifera*)	Anti-inflammatory and immunomodulatory potential	Clinical trials are required to establish the efficacy of herbal therapeutics in managing patients with COVID-19
11.	Traditional Chinese Medicine [167,168]	*Lian huaqing wen*	Inhibits SARS-CoV-2 replication and reduce the production of pro-inflammatory cytokines, TNF-α, IL-6, monocyte chemoattractant protein 1.	Majority of the available evidence are based on in vitro studies. Therefore, clinical trials are required to establish the efficacy
12.	Miscellaneous Immunomodulators [163,183]	BCG (Bacillus Calmette-Guérin)	BCG vaccine induces general effects on the function of immune system	The evidence for the protective effects of BCG is based on epidemiological data (COVID-19 mortality is negatively correlated with the extent of BCG vaccination). Therefore, clinical trials are required to establish the efficacy

* Not Applicable.

## Data Availability

Available data are presented in the manuscript.
